# Neurologically asymptomatic cerebral oligometastatic prostate carcinoma metastasis identified on [Ga]Ga-THP-PSMA PET/CT

**DOI:** 10.1186/s13550-020-00696-0

**Published:** 2020-09-22

**Authors:** M. I. Ross, N. Bird, I. A. Mendichovszky, Y. L. Rimmer

**Affiliations:** 1 School of Clinical Medicine, University of Cambridge, Cambridge Biomedical Campus, Hills Road, Cambridge, CB2 0QQ UK; 2 Department of Nuclear Medicine, Cambridge University Hospitals NHS Foundation Trust, Cambridge Biomedical Campus, Hills Road, Cambridge, CB2 0QQ UK; 3 Department of Radiology, University of Cambridge, Cambridge Biomedical Campus, Hills Road, Cambridge, CB2 0QQ UK; 4 Cancer Research UK Cambridge Centre, Cambridge Biomedical Campus, Hills Road, Cambridge, CB2 0QQ UK; 5 Department of Oncology, Cambridge University Hospitals NHS Foundation Trust, Cambridge Biomedical Campus, Hills Road, Cambridge, CB2 0QQ UK

**Keywords:** [^68^Ga]Ga-THP-PSMA, Prostate, Cancer, Carcinoma, Metastasis, Cerebral, Brain, Solitary, PET/CT

## Abstract

**Background:**

Brain metastases from prostate cancer are rare and usually only occur in the context of widespread systemic disease. This is the first case report of a solitary brain oligometastasis, in a neurologically intact prostate cancer patient with no other systemic disease, detected using [^68^Ga]Ga-THP-PSMA PET/CT and only the second one using a PSMA-based radiopharmaceutical.

**Case presentation:**

We report the case of a prostate cancer patient presenting 5 years after robot-assisted laparoscopic prostatectomy with biochemical recurrence, no neurological symptoms, and in the absence of metastatic lesions in the body on conventional imaging. A solitary cerebral metastasis was detected using [^68^Ga]Ga-THP-PSMA PET/CT, surgically resected, leading to a drop in serum PSA and a good recovery.

**Conclusion:**

In this case, [^68^Ga]Ga-THP-PSMA PET/CT resulted in a major change in clinical management and avoided additional morbidity associated with delayed diagnosis and treatment. This report demonstrates the importance of considering the presence of metastatic disease outside the conventional locations of prostate cancer spread, as well as the importance of ensuring comprehensive [^68^Ga]Ga-PSMA PET/CT coverage from vertex to upper thighs.

## Background

Prostate cancer usually metastasises to the pelvic lymph nodes and axial skeleton. Prostate cancer brain metastases mostly present in the late stages of disease and occur in an estimated 0.63% of patients [1]. The incidence of brain-only metastases in prostate cancer, in the absence of widespread metastatic disease, has been estimated from large cohorts of patients at 0.0061–0.2% [1–3]. Due to the low incidence of cerebral metastatic disease, cross-sectional neurological imaging is not routinely performed in prostate cancer patients and PET/CTs using PSMA-based radiopharmaceuticals are protocoled to cover the body from skull base to proximal thighs [4, 5]. Twenty-one case reports of solitary brain metastases from prostate cancer were found in the literature, summarised in Table 1 [6–21], adapted from Barakat et al. (2016) [17].

**Table 1:**
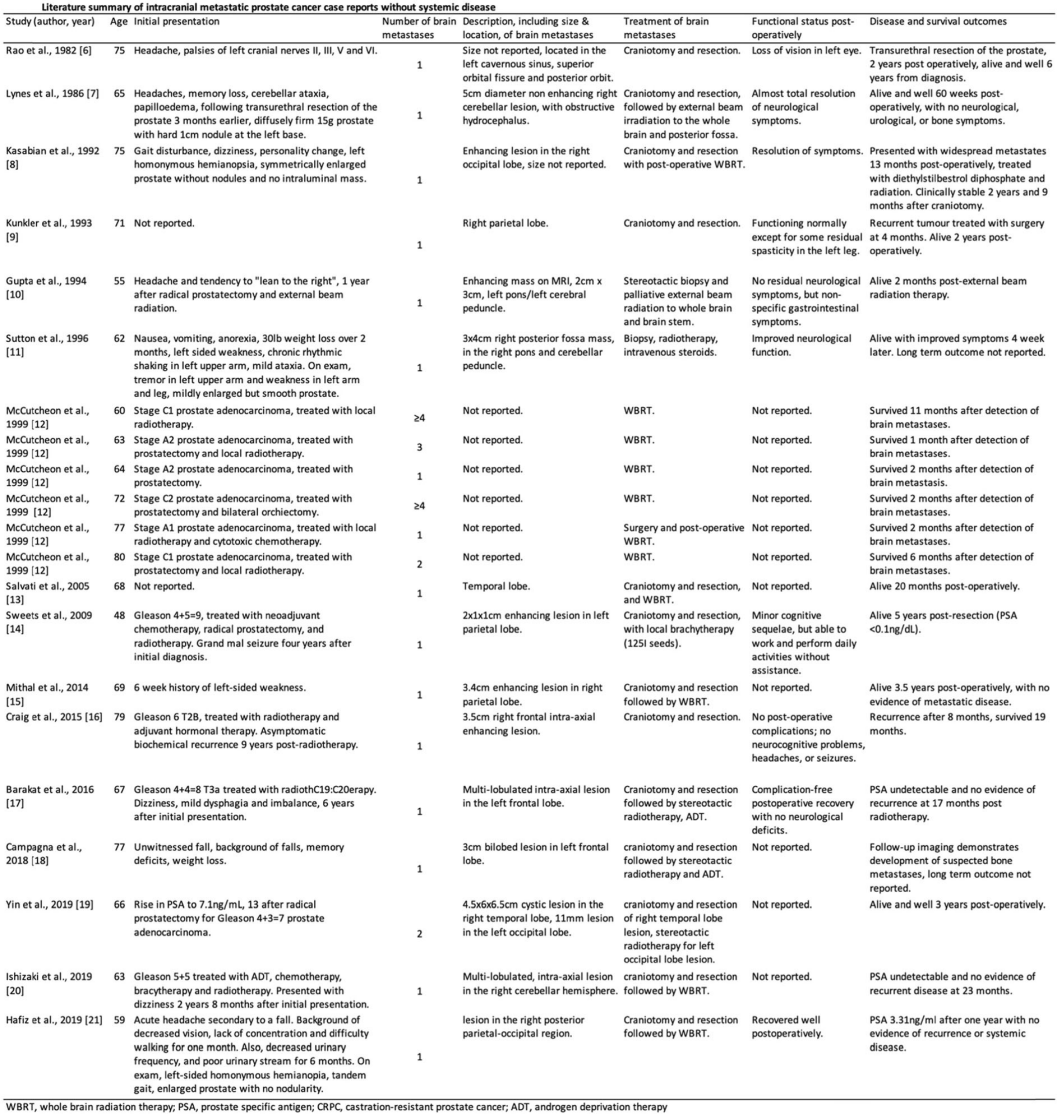
Literature summary of intracranial metastatic prostate cancer case reports without systemic disease

## Case presentation

**Fig. 1:**
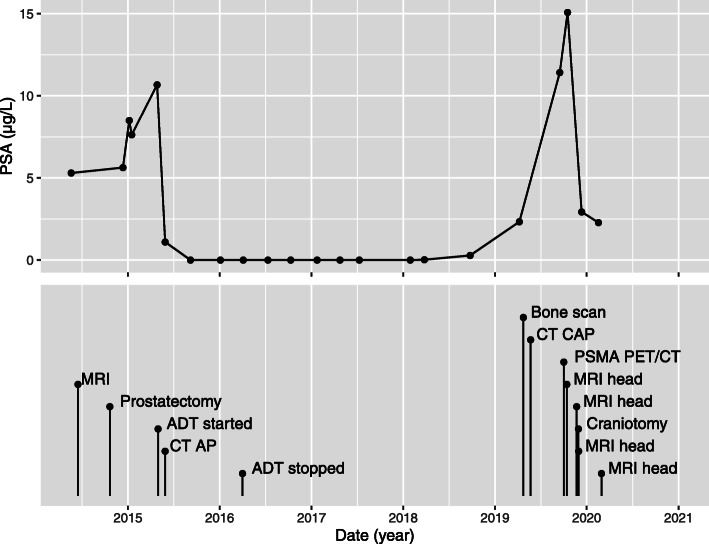
Serum PSA, with procedures, over time. Top: serum PSA (μg/L) over time (values of < 0.02 μg/L are plotted as 0 μg/L); bottom: procedures over time

A 62-year old male patient had a robot-assisted laparoscopic prostatectomy in August 2014 for a Gleason 4 + 3 multifocal adenocarcinoma with tertiary grade 5 disease and extraprostatic extension (pT3aN0Mx). The pre-operative PSA was 5.3 μg/L and the post-operative PSA was unchanged at 5.63 μg/L, despite negative surgical margins (Fig. 1). The PSA, 6 months post-operatively, had increased to 10.06 μg/L. Pelvic MRI showed no local recurrence or residual prostatic tissue and a bone scan was also negative. Androgen deprivation therapy (ADT) was commenced with a subsequent fall in PSA to < 0.02 μg/L, but it was discontinued after a year due to side effects.

Serum PSA tests were undertaken regularly (Fig. 1). 4.5 years post-operatively, the PSA increased to 2.33 μg/L and staging CT and whole-body bone scintigram showed no evidence of metastatic disease.

Five years after prostatectomy, the PSA reached 10.6 μg/L, and the patient underwent a [^68^Ga]Ga-THP-PSMA PET/CT using a cold kit PSMA formulation [22–25]. Sixty minutes after intravenous injection of 204 MBq of tracer a [^68^Ga]Ga-THP-PSMA PET/CT was acquired from proximal thighs to skull base on a GE Discovery 690 PET-CT scanner (GE Healthcare, Chicago, USA) according to current guidelines [5] (4 min per bed position with 23% overlap and axial field of view of 16 cm). A low-dose unenhanced CT scan (120 kVp, 0.5 s rotation time, 3.75 mm slices, Noise Index 45, 10–180 mA) was performed for attenuation correction and localization purposes. PET reconstructions included corrections for radiotracer decay, attenuation, modeled scatter, randoms and dead-time, both for time-of-flight (TOF ordered subset expectation maximisation with 24 subsets and 2 iterations) and scatter-limit correction series. The PET/CT showed increased focal uptake in the right mid-cranial fossa, with no focal uptake in the rest of the body. The differential considered at this point was a meningioma, glioma or a prostate metastasis, all known to express PSMA [26–29]. The patient had no neurological symptoms at the time of presentation.

**Fig. 2:**
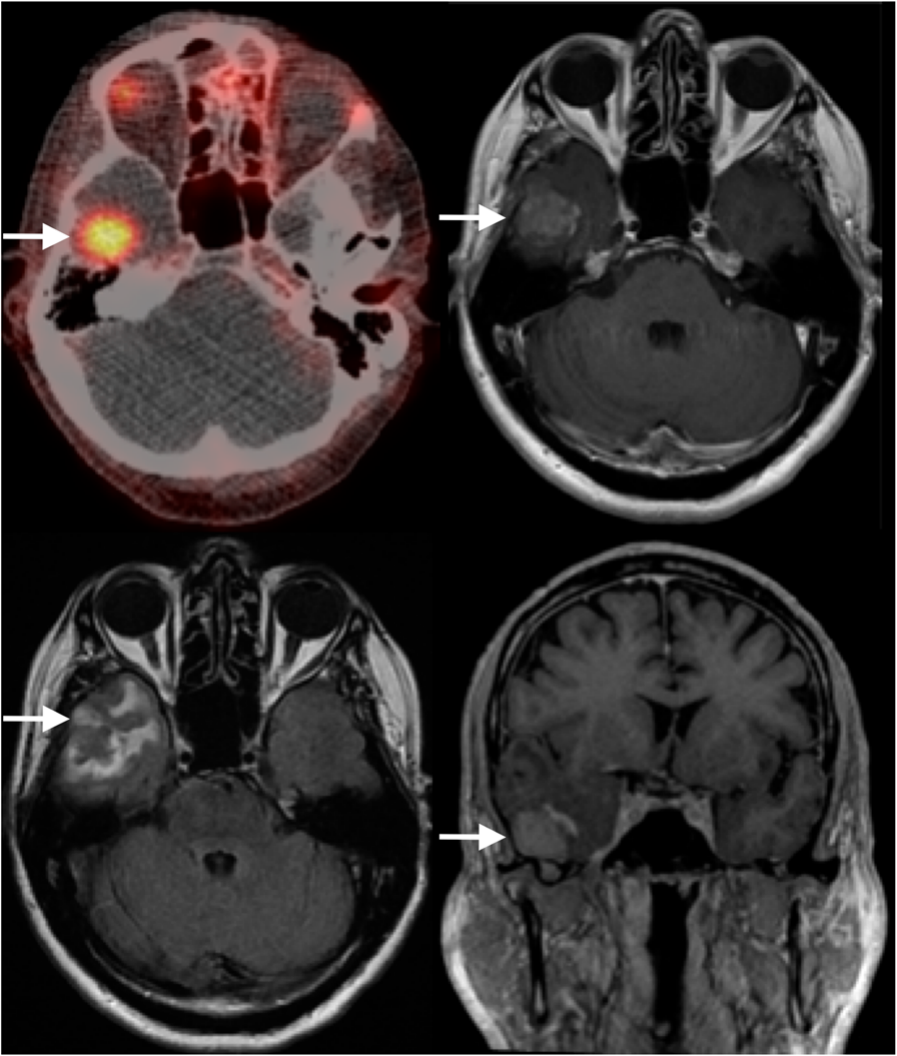
Selected images through the skull base from the [^68 Ga]Ga-THP-PSMA PET/CT and subsequent MRI. Top left: axial fused PET/CT image revealing focal uptake in the right middle cranial fossa; bottom left: axial FLAIR MRI showing white matter oedema in the right temporal lobe; top (axial) and bottom (coronal) right: post-contrast enhanced T1-weighted MR images demonstrating an enhancing lesion in the floor of the right middle cranial fossa in close contact with the dura mater

A subsequent contrast-enhanced MRI head showed a well-demarcated enhancing dural-based lesion in the floor of the right middle cranial fossa, projecting into the right temporal lobe, with surrounding T2 hyperintense changes in the right temporal lobe, believed to be extra-axial on MRI. The lesion was thought initially to represent a meningioma, both on the [^68^Ga]Ga-THP-PSMA PET/CT (SUV_max_ TOF = 6.2) and contrast-enhanced MRI, although the PET/CT had raised the suspicion of a prostate cancer metastasis given its unusually intense uptake (Fig. 2).

As uncertainty remained between the two possible diagnoses (meningioma or prostate cancer metastasis) in the neuro-oncology multidisciplinary team meeting, the patient was referred for a neurosurgical opinion, which advised the lesion’s surgical resection and dexamethasone 4 mg bd, to reduce surrounding oedema. A second MRI head, performed 1 month later for pre-surgical planning, confirmed a 23 × 18 mm solitary tumour with surrounding oedema in the anterior part of the right inferior temporal gyrus. The tumour contained heterogeneous low and high areas of T2 signal, patchy heterogeneous contrast enhancement and dural extension to the brain surface inferiorly. The second MRI suggested that the lesion was intra-axial. The contrast-enhanced post-operative MRI head showed complete resection of the tumour with a reduction in serum PSA to 2.93 μg/L. The histopathology report confirmed an intra-axial right temporal lobe metastatic prostatic adenocarcinoma with dural extension and intense PSA and PSAP staining. Three months after neurosurgery the patient had recovered well with only mild neurological deficit (left foot drop). Surveillance MRI showed no intracranial mass or abnormal contrast enhancement to indicate residual or recurrent tumour and only expected post-operative encephalomalacia in the anterior inferior part of the right temporal lobe. The serum PSA remained elevated (2.28 μg/l). Currently, the patient is followed up with 3-monthly MRIs and PSA monitoring. ADT has not been restarted. Although the post-operative MRI head showed complete resection, the fact that histopathology documented dural involvement could explain the elevated PSA after surgery and potential presence of residual microscopic meningeal disease. The patient is currently asymptomatic, but he will undergo further PSMA PET/CT if the PSA continues to rise.

## Discussion

This is the first case report of a solitary brain oligometastasis in a neurologically intact prostate cancer patient with no other systemic disease using [^68^Ga]Ga-THP-PSMA PET/CT and only the second one using a PSMA-based radiopharmaceutical [19]. Current guidelines for [^68^Ga]Ga-PSMA PET in prostate cancer patients recommend skull base to proximal thighs coverage [5], which may result in brain or skull metastases located more cranially remaining undetected. In our case, the solitary brain metastasis was only seen on the most cranial PET slices, and, left undiagnosed, would have further increased in size until neurological symptoms became apparent, resulting in delayed treatment and increased morbidity. Our report illustrates the need to consider the presence of metastatic disease outside the conventional locations of prostate cancer in patients with persistent or rising PSA and negative conventional imaging.

The other case report, from Yin et al. (2019), describes a clinical situation remarkably similar to ours [19]. Both report asymptomatic patients who presented with a rise in serum PSA, after having previously undergone radical prostatectomy for Gleason grade 4 + 3 = 7 prostate adenocarcinoma. Both patients were subsequently found to have a cerebral metastasis in the right temporal lobe, although the patient in Yin et al. (2019) also had a smaller metastasis in the left occipital lobe.

The use of [^68^Ga]Ga-PSMA PET/CT played a crucial role in the management of these patients and led to detection and treatment of their disease. A recent study [30] found that in patients with biochemical recurrence and in the absence of radiological evidence of metastatic disease on CT or bone scan [^68^Ga]Ga-PSMA PET/CT may lead to a change in management for up to 96% of patients. Although this study is limited by a small number (33) of patients, the results are nonetheless impressive. For our patient, the [^68^Ga]Ga-THP-PSMA PET/CT resulted in a major change in management (surgical resection) and prevented a delayed presentation with neurological symptoms, systemic (including further intracranial) metastatic disease, emergency neurosurgery and increased morbidity.

## Conclusion

This is the first documented case of a solitary cerebral metastasis from prostate cancer in the absence of systemic disease being diagnosed using [^68^Ga]Ga-THP-PSMA PET/CT. It demonstrates the usefulness of this relatively new imaging modality in the investigation and staging of prostate cancer, as well as the importance of ensuring comprehensive, vertex-to-thighs, coverage when performing a [^68^Ga]Ga-PSMA PET study in patients with high suspicion of prostate cancer metastases.

## Data Availability

All data generated or analysed during this study are included in this published article.

## Abbreviations

[^68^Ga]Ga-PSMA: [^68^Ga]Ga-prostate-specific membrane antigen
[^68^Ga]Ga-THP-PSMA: [^68^Ga]Ga-trishydroxypyridinone (THP)-prostate-specific membrane antigen (PSMA)
ADT: Androgen deprivation therapy
CT: Computed tomography
MRI: Magnetic resonance imaging
PET: Positron emission tomography
PSA: Prostate-specific antigen
PSMA: Prostate-specific membrane antigen
