# Correction to: Neurologically asymptomatic cerebral oligometastatic prostate carcinoma metastasis identified on [Ga]Ga-THP-PSMA PET/CT

**DOI:** 10.1186/s13550-020-00719-w

**Published:** 2020-10-27

**Authors:** M. I. Ross, N. Bird, I. A. Mendichovszky, Y. L. Rimmer

**Affiliations:** 1 School of Clinical Medicine, University of Cambridge, Cambridge Biomedical Campus, Hills Road, Cambridge, CB2 0QQ UK; 2 Department of Nuclear Medicine, Cambridge University Hospitals NHS Foundation Trust, Cambridge Biomedical Campus, Hills Road, Cambridge, CB2 0QQ UK; 3 Department of Radiology, University of Cambridge, Cambridge Biomedical Campus, Hills Road, Cambridge, CB2 0QQ UK; 4 Cancer Research UK Cambridge Centre, Cambridge Biomedical Campus, Hills Road, Cambridge, CB2 0QQ UK; 5 Department of Oncology, Cambridge University Hospitals NHS Foundation Trust, Cambridge Biomedical Campus, Hills Road, Cambridge, CB2 0QQ UK

## Correction to: EJNMMI Res (2020) 10:108 https://doi.org/10.1186/s13550-020-00696-0

Following publication of the original article [1], the authors have reported that there are two errors concerning the representation of the PET imaging agent ‘[^68^Ga]Ga-THP-PSMA PET/CT’; one error affects the article title, and the other affects the caption of Fig. 2.

In the article title, the agent is (incorrectly) written as ‘[Ga]Ga-THP-PSMA PET/CT’, while in the caption of Fig. 2 it is (incorrectly) written as ‘[^68 Ga]Ga-THP-PSMA PET/CT’.

Please note that the correct representation is ‘[^68^Ga]Ga-THP-PSMA PET/CT’.

The authors apologize for any inconvenience caused.
